# Functionality of bacterial communities in constructed wetlands used for water purification: influence of root components and seasonality

**DOI:** 10.3389/fpls.2025.1480099

**Published:** 2025-02-11

**Authors:** Yao Huang, Weili An, Tianzhu Ning, Zhiguang Ma, Yuelin Li, Ke Liu, Lingbo Ji, Hongxiao Liu, Dafeng Hui, Hai Ren

**Affiliations:** ^1^ Guangdong Provincial Key Laboratory of Applied Botany, South China Botanical Garden, Chinese Academy of Sciences, Guangzhou, China; ^2^ School of Ecology, Hainan University, Haikou, China; ^3^ College of Horticulture and Landscape Architecture, Zhongkai University of Agriculture and Engineering, Guangzhou, China; ^4^ China State Construction Engineering Cooperation, Lanzhou, China; ^5^ Ecology and Environmental Science Research & Design Institute of Zhejiang Province, Hangzhou, Zhejiang, China; ^6^ Department of Biological Sciences, Tennessee State University, Nashville, TN, United States

**Keywords:** bacterial communities, root components, water purification, seasonality, constructed wetland

## Abstract

**Introduction:**

Constructed wetlands have become crucial ecosystems for the purification of industrial and agricultural water. The health of wetland plants and the efficacy of water purification are strongly influenced by root-associated bacteria. However, our understanding of the functions of bacterial communities in the plant different root components (i.e., rhizosphere, rhizoplane, and endosphere) and their impact on water purification is still limited.

**Methods:**

To address this knowledge gap, we employed high-resolution 16S rRNA deep amplicon sequencing to explore the bacterial community structure and assembly within the root components of three plant species (i.e. *Iris ensata*, *Canna indica*, and *Hymenocallis littoralis*) found in constructed wetlands.

**Results:**

Our findings revealed that the pollutant removal efficiency was higher in the wet season than in the dry season. The specific root compartment, plant species, environmental factors, and seasonality significantly influenced the bacterial composition, diversity and abundance. Across all three plant species, Proteobacteria emerged as the dominant bacterial groups in all root components. The abundance and diversity of bacterial communities exhibited a decline from the rhizosphere to the endosphere, accompanied by an increase in the number of distinctive biomarkers from the rhizosphere to the endosphere. The bacterial composition exhibited significant similarity in the rhizosphere in the dry season and the endosphere in the wet season. Bacterial genes in the rhizosphere-rhizoplane were associated with environmental information processing, transportation and metabolism, while those in the rhizoplane-endosphere primarily handle metabolic processes. The bacterial community positively correlated with total nitrogen content, chemical oxygen demand, and NO_4_^+^-N in the dry season, while associated with total phosphorus, total organic carbon, and NO_3_^+^-N content in the wet season.

**Discussion:**

The structure and function of the bacterial community within the root rhizoplane-endosphere can serve as indicators of the water purification efficacy of constructed wetlands.

## 1 Introduction

Water pollution has become a serious environmental problem worldwide, including China (Beach, 2001; Teurlincx et al., 2019). Wastewater often constitutes a significant source of effluents released into agriculture, rivers, and oceans, carrying substantial loads of water and fecal bacteria (Mathavarajah et al., 2020). Constructed wetlands have proven to be highly efficient and cost-effective ecotechnologies that harness natural processes involving wetland plants, soil, and their associated microbial to effectively treat water (Liu et al., 2008; Vymazal, 2014). Constructed wetland ecosystems play a multifaceted role in environmental preservation by reducing the levels of nitrogen (N), phosphorus (P), and organic matter in water, all while creating unpolluted landscapes (Thurston et al., 2001; Guo et al., 2015). Consequently, many countries have either established or are in the process of constructing such wetlands to enhance wastewater quality and decrease contaminants (Imfeld et al., 2009; Wu et al., 2015). The combination of plants, substrate and microorganisms collectively achieves a remarkable 90% removal efficiency of TN, SO_2_ (Makgato and Chirwa, 2020; Yu et al., 2024). The effectiveness of constructed wetlands in water purification varies significantly among different plant species, and this discrepancy is closely intertwined with the role of root microorganisms (Brisson and Chazarenc, 2009). Microbial communities residing in plant roots are important to biogeochemical processes within wetlands, influencing the dynamics of soil nutrients (e.g., N and P) and, in turn, the ecological functions of constructed wetlands (Sims et al., 2012). Root microorganisms are most important for microbial communities. Different root-associated components (i.e., the rhizosphere, rhizoplane and endosphere) was found to harbor a distinct microbiome (Chen et al., 2016). However, so far there is limited knowledge concerning the structure and functions of plant root-associated microbiomes in the context of water purification in constructed wetlands.

Microorganisms often perform vital roles in nutrient management and pollution controls (Montreemuk et al., 2023), e.g., bacteria help reduce N levels by enhancing nitrification-denitrification processes in wastewater (Liu et al., 2019; Lu et al., 2020). Some dominant rhizoplane and endosphere bacteria can potentially affect the carbon (C), N, and P cycles in wetland ecosystems (Liao et al., 2018). The methanotrophs have important role in methane removal in wetlands, which slow down the greenhouse effect (Wang et al., 2023). The effects of composition of bacteria community are important to the water purification.

Moreover, different root component microorganisms have different capacities in nutrient and pollution management in constructed wetlands. The ability to degrade pollutants was found to be greater for rhizosphere microorganisms than for non-rhizosphere microorganisms (Man et al., 2020). The rhizoplane bacteria may help remove organic matter from water-contaminated water (Kovacs et al., 1999; Saeed et al., 2016). In addition to their role in the degradation of organic pollutants, endophytic bacteria have been found to potentially enhance immune responses in plants and establishing a symbiotic relationship with them (Ali et al., 2024). Therefore, studying the potential function of microbial communities in different root components becomes essential in gaining a comprehensive understanding of the water purification capacity within wetland ecosystems.

To enhance the pollutant removal efficiency of constructed wetlands, prior research efforts have focused on various aspects, including the selection of appropriate plant species (Brisson and Chazarenc, 2009; Liu et al., 2016), the composition of root-associated bacterial communities, and their functions (Naylor et al., 2017). Numerous studies have consistently demonstrated that the presence of vegetation substantially enhances the abundance of microbial communities in comparison to wetlands lacking vegetation (Pang et al., 2016; Wang et al., 2021). Moreover, wetland plants have been shown to mitigate methane emissions from wetlands by creating a habitat conducive to methane oxidation (Wang et al., 2023). Simultaneously, the composition and activities of root-associated microorganisms have a profound impact on the water purification capacity of constructed wetlands (Arroyo et al., 2013; Fu et al., 2006). It is essential to note that the roots of these plants serve as the primary interface for plant-microbe interactions, providing essential substrates to support microbial activities (Zhang et al., 2017). The interplay between plant species and root components (rhizosphere and endosphere) in shaping microbial communities represents a collaborative synergy (Chen et al., 2020; Sun et al., 2021). Distinct plant species have been observed to exert significant influence on both structural and functional characteristics of rhizosphere microbial communities in constructed wetlands (Man et al., 2020). Multiple studies have demonstrated that the presence of plants significantly increases the abundance of microbial communities compared to their absence (Pang et al., 2016; Wang et al., 2021). Moreover, the abundance of microbial communities in the rhizosphere soil of a grassland was found to be more than two-fold greater than in the non-rhizosphere soil (Balasooriya et al., 2012). Intriguingly, variations in microbial communities within different root components of a single plant species have been documented (Edwards et al., 2015). Similarly, akin to the rhizosphere, the rhizoplane has been found to host a diverse array of bacterial communities (Rifaat et al., 2002). This comprehensive exploration of distinct microbiome structures and functions within various root-associated components holds promise as an indicator of the water purification capacity within wetland ecosystems.

In this study, we assessed the bacterial communities in the rhizosphere, rhizoplane, and endosphere of three plant species that are commonly planted in constructed wetlands in South China. We attempted to answer the following questions: (1) Which plant species exhibited the highest water purification capacity? (2) Did the characteristics and potential functions of bacterial communities differ among different root components (rhizosphere, rhizoplane, and endosphere) and plant species? and (3) How did the main bacterial communities of different root components affect the water purification process of constructed wetlands?

## 2 Materials and methods

### 2.1 Site description and sampling

The research was conducted in the Xiashan constructed wetland near the Pingshan River in Shenzhen, Guangdong Province, China (114.34°E, 22.69°N). Xiashan is a subsurface constructed wetland with a treatment scale of 31000-40000 m^3^/d, and the influent of the wetland is the tail water of the Shangyang Wastewater Treatment Plant. The following plants had been planted and were growing well in the wetland: *I. ensata*, *C. indica*, *H. littoralis*, *Thalia dealbata*, *Cyperus papyrus*, and *Cyperus alternifolius*. From top to bottom, the substrate of the wetland consisted of A-type filler with a thickness of 900 mm (slow-release C source: activated C: oyster shell: Zeolite: sand = 1:3.9:10.95:33.15:114.91); crushed stone (diameter: 4-8 mm) with a thickness of 300 mm; and crushed stone (diameter: 16-32 mm) with a thickness of 300 mm. At the time of this research, the wetland system had been running stably and continuously for nearly 2 years and had provided substantial water purification (Gao et al., 2020). The area has a south subtropical monsoon climate and the annual temperature ranging from 5.3 - 36.6°C in 2021.

Three plant species (i.e., *H. littoralis*, *I. ensata*, and *C. indica)* were selected, because they have been shown to have good contaminant removal capabilities (Singh et al., 2025). In 2021, we sampled three wetland ponds with *H. littoralis*, *I. ensata*, and *C. indica* in January (i.e. dry season), and sampled five wetland ponds with the same three species in July (i.e. wet season). Each pond covered an area of approximately 1245 m^2^. Plant roots were not contaminated by bulk soil in the constructed wetland, so we uprooted softly three plant samples at the five locations (the four corners and the center) at each pond, and the corresponding soil samples were collected using an ethanol sterilized shovel. After the mixed samples were placed in sterile Ziploc bags, they were transported to the laboratory. We then gently scrapped off a thin layer of soil (sand, 0-5 mm) from the roots and mixed the samples from the five locations to obtain one sample of rhizosphere soil per pond (Pei et al., 2018). Rhizoplane material was separated from plant roots after washing 3x in sodium-free phosphate buffer and filtered with a 0.22 µm polycarbonate filter prior to DNA extraction. The endosphere was represented by the remaining root material, i.e., roots minus rhizosphere soil and rhizoplane. Each sample of rhizosphere soil or rhizoplane and endosphere root material was represented by about 5 g of material.

As indicated, we collected three samples of the rhizosphere, rhizoplane, and endosphere from each of the three ponds for each of the three plant species. This yielded 27 samples: 3 plant species × 3 ponds/plant species × 3 root components (rhizosphere, rhizoplane, and endosphere)/pond/plant species. The samples were stored at -80°C and were then sent to Beijing Biomarker Biotechnology Co., Ltd. under low temperature conditions for microbial high-throughput sequencing.

### 2.2 DNA extraction, PCR amplification, and Illumina MiSeq sequencing

A soil DNA extraction kit (MN NucleoSpin 96 Soi) was used to extract DNA from rhizosphere, rhizoplane, and endosphere samples of the three plant species. The V3+V4 region of the bacterial 16S rRNA gene was amplified using primers 338F/806R (5’-ACTCCTACGGGAGGCAGCA-3’/5’-GGAC-TACHVGGGTWTCTAAT-3’), and endophytic universal primers 335F/769R (5’-CADACTCCTACGGGAGGC-3’/5’-ATCCTGTTTGMTMCCCVCRC-3’). PCR reactions were carried out with 50 ng/uL ± 20% of genomic DNA, 5 μL of KOD FX Neo Buffer, 0.2 μL of KOD FX Neo, 2 μL of 2dNTP, and sufficient ddH_2_O to increase the total volume to 10 μL. The PCR included an initial denaturation at 95°C for 5 min; followed by 25 cycles of 95°C for 30 s, 50°C for 30 s, and 72°C for 40 s; and a final extension at 72°C for 7 min. After 1.8% agarose gel electrophoresis (120 V for 40 min), the target fragment was cut and recovered. The products were purified, quantified, and homogenized to form a sequencing library. The library was first inspected for quality, and the qualified library was subjected to bidirectional sequencing using an Illumina HiSeq 2500 (Wright and Vetsigian, 2016).

The original data were spliced (FLASH, version 1.2.11) (Magoc and Salzberg, 2011), and the spliced sequences were filtered by quality (Trimmomatic, version 0.33) (Bolger et al., 2014). The chimera (UCHIME, version 8.1) were then removed to obtain high-quality tag sequences (Edgar et al., 2011). Sequences were clustered at a 97% similarity level (USEARCH, version 10.0) (Edgar, 2013) and then used 0.005% of all sequences sequenced as a threshold to filter OTU (Bokulich et al., 2013). Based on Silva SSU and LSU databases 138 (Quast et al., 2013), we annotated OTU using the RDP Classifier software (version 2.2, confidence threshold 0.8) (Wang et al., 2007) to derive the species classification for each OTU. We then counted OTUs to determine the community composition of each sample.

### 2.3 Water quality monitoring

The water streams are already filtered and passed through secondary treatment (e.g., aeration and clarification) before being discharged into the constructed wetland. Water samples were collected from the water inlet and outlet of the three ponds, three replicates were collected from each inlet and outlet. All of the water samples were transported to the laboratory for chemical analyses. Chemical oxygen demand (COD) was measured using a spectrophotometer (DR/2010, Hach Co., Loveland, CO, USA). Total nitrogen (TN), total organic carbon (TOC), NO_3_^−^-N, NO_2_^−^-N, NH_4_^+^-N, and total phosphorus (TP) were analyzed according to standard methods (APHA, 1998). For these pollutants, the removal efficiencies for each pond were calculated from the difference in concentration between the water inlet and outlet.

### 2.4 Statistical analysis

The OTU data were clustered at a 97.0% similarity level after standardization. The 10 most abundant genera in the root components of the three plant species were analyzed using R 3.2.5. The statistical power analysis has been conducted to ensure that the sample size is large enough in data analysis. The α diversity indices (ACE, Chao1, Shannon-Wiener) were calculated using Mothur version v.1.30 (http://www.mothur.org/) and SPSS21.0 software, and were visualized with Origin software (Shao et al., 2016). ACE and Chao1 indices were used to assess bacterial community richness. The Shannon-Wiener index was used to assess bacterial community diversity. To investigate the patterns of bacterial community structure, β diversity of the bacteria was assessed. We also performed nonmetric multidimensional scaling (NMDS) with the weighted_UniFrac distance calculated from the OTU community matrix (Mitter et al., 2017). Biomarker analysis with linear discriminant analysis effect size (LEfSe) (http://huttenhower.sph.harvard.edu/lefse/) was used to identify bacteria abundance differed among plant species and root components (Chen et al., 2020). After that, PICRUSt (phylogenetic investigation of communities by reconstruction of unobserved states) was used to predict the potential functions of the OTUs (relative abundance > 1%) in the rhizosphere vs. the rhizoplane and in the rhizoplane vs. the endosphere based on the KEGG (Kyoto Encyclopedia of Genes and Genomes) database (Langille et al., 2013). Redundancy analysis (RDA) was then performed using CANOCO 5.0 (Microcomputer Power, Ithaca, NY, USA) to assess the relationships between water physiochemical properties and species composition of biomarkers (Morris and Blackwood, 2015).

## 3 Results

### 3.1 Water purification efficiency in three plant species

**Figure 1:**
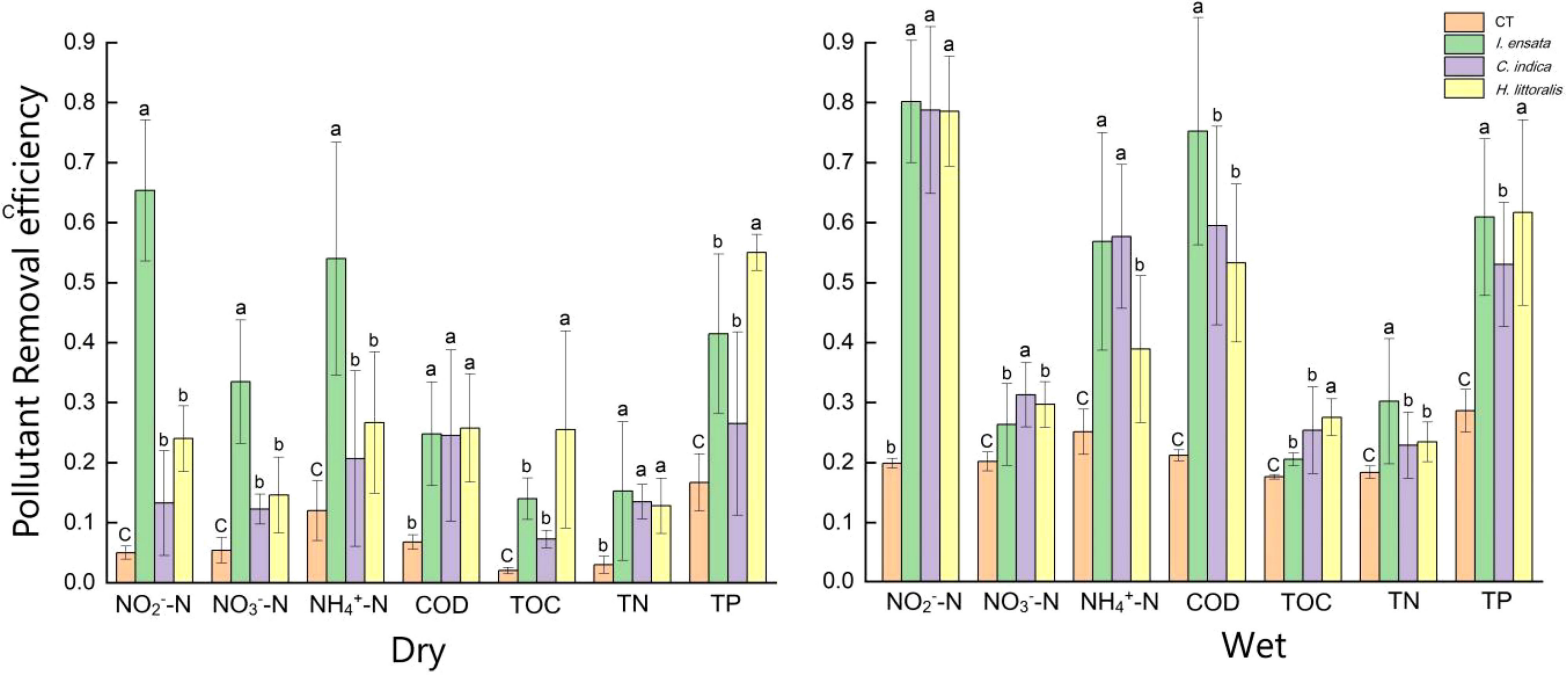
Water purification efficiency of three kinds of plants in different seasons. CT, Control treatment; Dry, dry season; Wet, wet season. The a, b, c means represent whether there is a significant difference in the environmental factors of different plants.

The pollutant removal efficiencies of three plant species varied significantly between two seasons (**Figure 1**). During the wet season, plants exhibited heightened pollutant removal capacities compared to the dry season. Surprisingly, the pollutant removal efficiencies of *I. ensata* to purify NO_3_^−^-N weakened in the wet season. In the dry season, *I. ensata* exhibited significantly higher pollutant removal efficiencies for NO_2_^−^-N, NO_3_^−^-N, and NH_4_^+^- N compared to the other two plant species. In the wet season, *I. ensata* had the highest pollutant removal efficiency for COD and TN, while *C. indica* had the highest pollutant removal efficiency for NO_3_^−^-N. *H. littoralis* had a significantly higher pollutant removal efficiency for TOC and TP than the other two plant species in both seasons.

### 3.2 Characteristics of bacterial communities of different root components in three plant species

**Figure 2:**
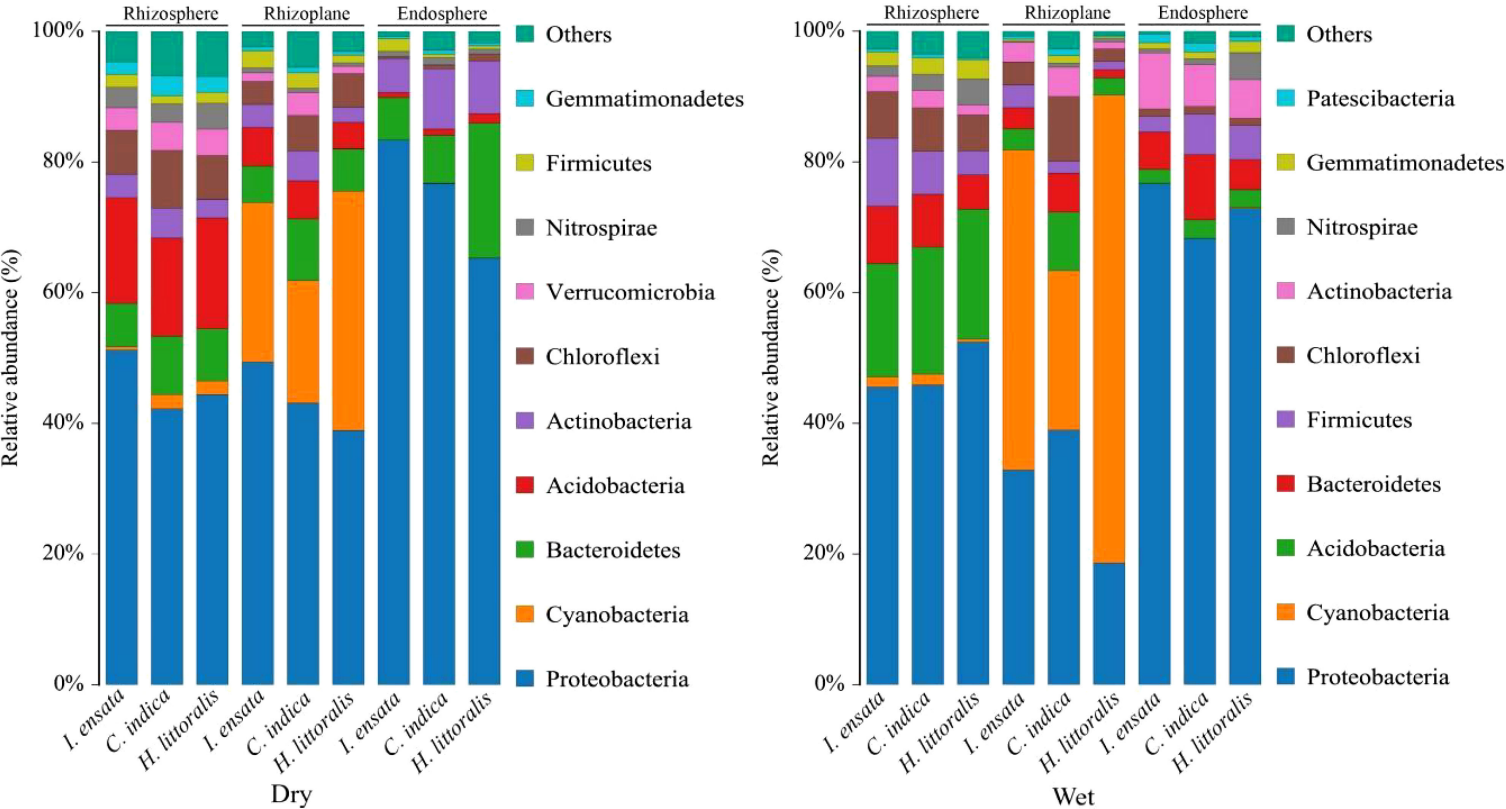
Dominant groups (relative abundance > 0.1%) of bacteria at the phylum level in the rhizosphere, rhizoplane, and endosphere of three plant species in the dry and wet seasons, respectively. Dry, Dry season; Wet, Wet season.

At the phylum level, significant differences were observed in the bacterial composition among different root components and between two seasons (**Figure 2**). Proteobacteria had the highest relative abundance in the root among three plant species (over 40% except in the rhizoplane in the wet season). Alphaproteobacteria and Gammaproteobacteria were the predominant bacteria within the Proteobacteria phylum among the three plant species, collectively constituting an average relative abundance as high as 50% (**Supplementary Figure S1**). Caynobacteria, Bacteroidetes and Acidobacteria were also had large relative abundance. Acidobacteria had the second largest relative abundance in the Rhizosphere, Caynobacteria had the second largest relative abundance in the Rhizoplane, while Bacteroidetes had the second largest relative abundance in the Endosphere in the dry season. Bacteroidetes had the second largest relative abundance in the Rhizosphere, and Acidobacteria had the second largest relative abundance in the Endosphere in the wet season. Caynobacteria of the Rhizoplane increased significantly in the wet season than that in the dry season.

**Figure 3:**
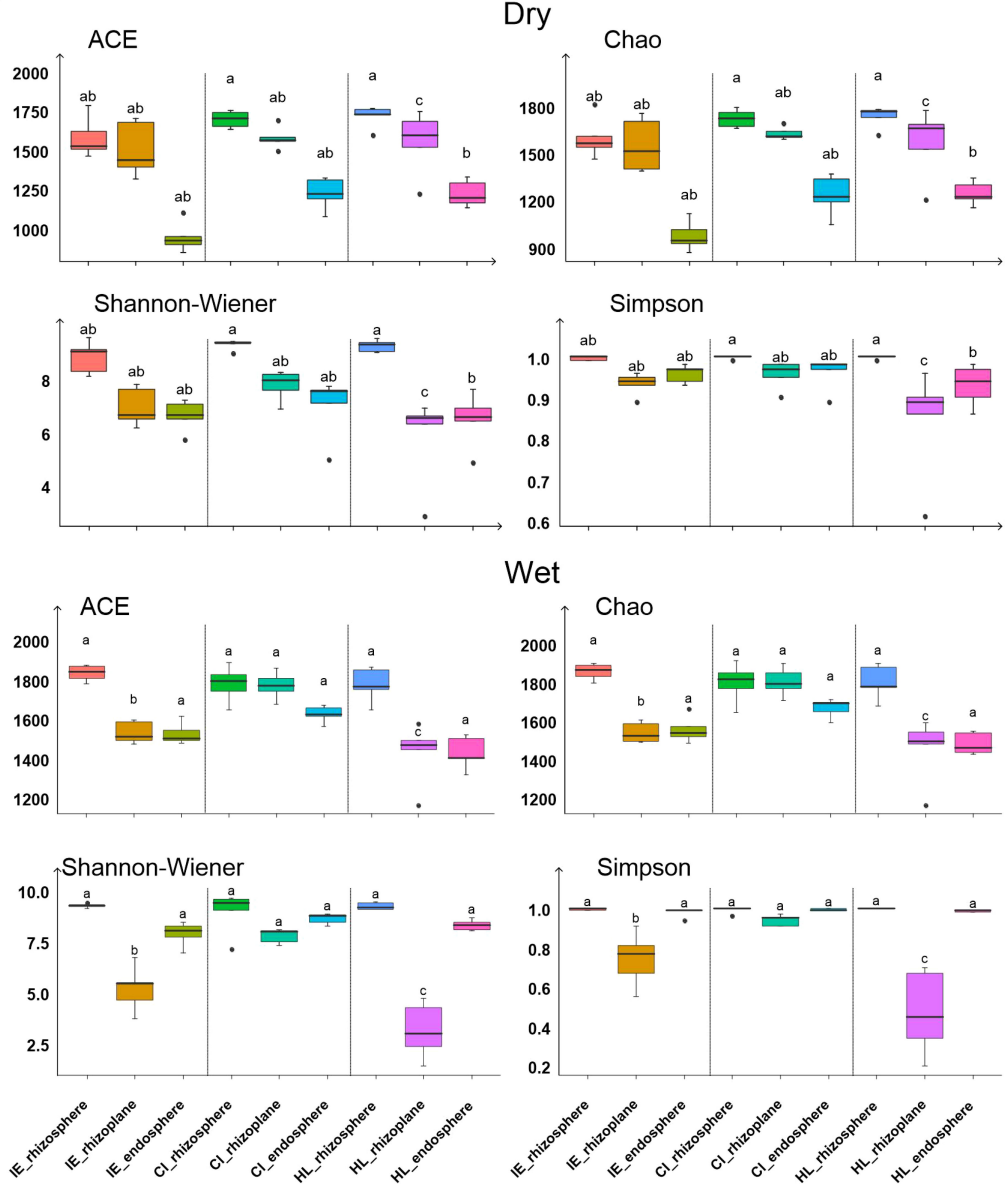
The α diversity of bacteria in the root components of the three plant species. Values are means ± SE. Within each panel and each root compartment, means with different letters are significantly different. IE, *I. ensata*; CI, *C. indica*; HL, *H. littoralis*; Dry, dry season; Wet, wet season.

For each of the three plant species, the α-diversity of bacterial communities decreased from the rhizosphere to the rhizoplane and further to the endosphere (**Figure 3**). In the rhizosphere, taxon richness, as indicated by ACE and Chao1 indices, was significantly higher for *C. indica* and *H. littoralis* than for *I. ensata* (*p*<0.05), although the Shannon-Wiener diversity index did not significantly differ among the plant species. On the rhizoplane, the Shannon-Wiener diversity index was significantly higher for *C. indica* compared to *H. littoralis* and *I. ensata* (*p*<0.05). In the endosphere, the ACE and Chao1 indices were significantly higher for *C. indica* and *H. littoralis* than for *I. ensata* (*p*<0.05) (**Figure 3**). Additionally, the diversity and abundance of bacterial community was higher in the wet season than in the dry season.

**Figure 4:**
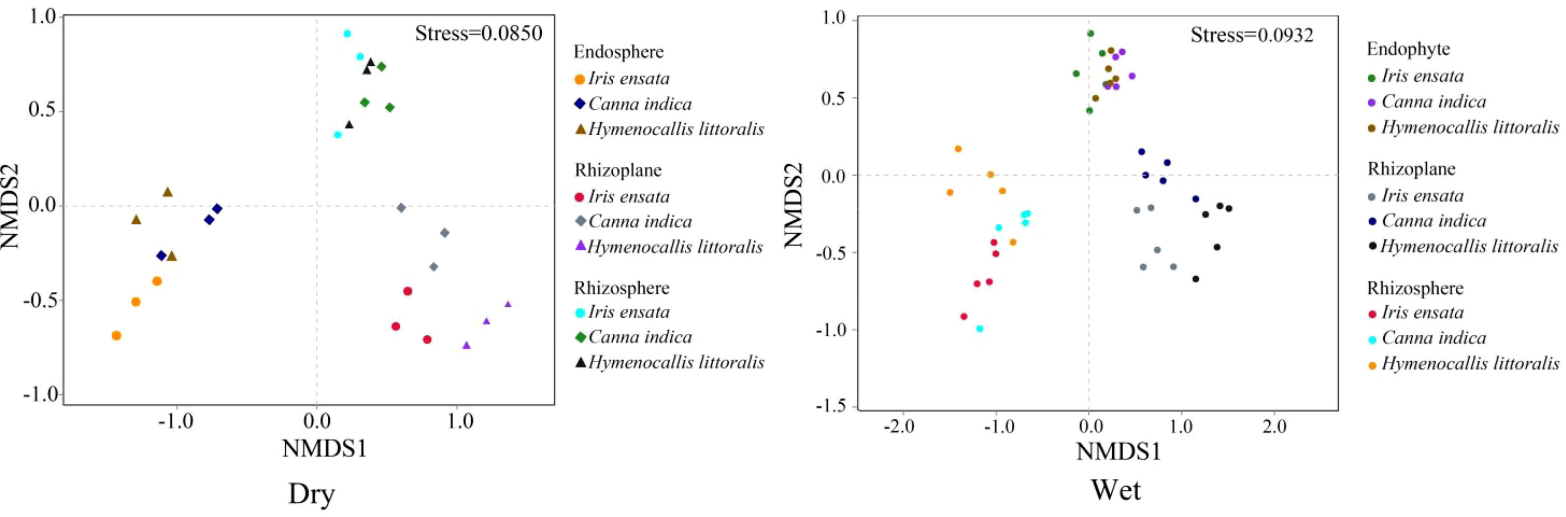
Comparison of annual bacterial communities in the three root components of the three plant species as indicated by nonmetric multidimensional scaling analysis (NMDS) of the weighted unifrac distances. Dry, dry season; Wet, wet season.

The β diversities of bacterial communities, assessed by Bray-Jaccard dissimilarity, showed distinct variations among the three root components across different species and seasons (**Figure 4**). Similarity in bacterial communities was observed within the same root compartment across different plant species, while differences in the composition of bacterial community were evident among the three components within the same plant species. The rhizosphere had the largest overlapping region, followed by the endosphere, and the smallest overlap was observed in the rhizoplane. This pattern indicates that the similarity of bacteria was highest for the rhizosphere, intermediate in the endosphere, and lowest in the rhizoplane.

### 3.3 Identification of bacterial biomarkers of different root components in three plant species

Different bacterial biomarkers were identified between various plant species and different root components (**Supplementary Figure S1**). The taxonomic hierarchy, ranging from phylum to genus, was represented by concentric rings (**Supplementary Figures S1A–C**). Using an LDA score threshold of 3.0, the number of discriminative biomarkers among the three plants tended to increase from the rhizosphere (13 clades) to the rhizoplane (24 clades) and further to the endosphere (29 clades). In the case of *I. ensata*, the number of bacterial biomarkers in the rhizosphere, rhizoplane, and endosphere was 1, 0, and 5, respectively. These numbers were 10, 21, and 12 for *C. indica*, and 2, 3, and 12 for *H. littoralis*. Notably, the number of biomarkers in bacterial communities was highest on the rhizoplane of *C. indica* and lowest for of *I. ensata*. Specific bacterial taxa with the highest relative abundances in different root components included Oxyphotobacteria (LDA = 4.06) in the rhizosphere of *C. indica*, Deltaproteobacteria (LDA = 4.26) on the rhizoplane of *C. indica*, Caulobacterales (LDA = 4.44) in the endosphere of *I. ensata*, and Devosiaceae (LDA = 4.18) in the endosphere of *C. indica.*

### 3.4 Potential functions of bacterial communities in the root components of the three plant species

The PICRUSt analysis identified a total of eight gene families that relate to metabolic functions of the bacterial communities in both dry and wet seasons (**Supplementary Figures S2, S3**). Principal functional pathways, including metabolism, environmental information processing, and organic systems, were consistently detected as the main functional genes across all plant species in both seasons. Among these, metabolism-related genes were the most abundant. The metabolic functions reached their peak for *I. ensata* roots in the dry season (41.5 and 41.9, **Supplementary Figures S2A, D**) and for *H. littoralis* roots in the wet season (42.6 and 42.6) (**Supplementary Figures S3C, F**).

The plant species had a significant effect on the predicted functions of bacterial communities among root components in different seasons (**Supplementary Figures S2, S3**). Specifically, in the dry season, the abundance of genes related to metabolism was lower in the rhizosphere and rhizoplane across three plant species compared to the endosphere. Conversely, in the wet season, the relative abundance of genes related to metabolism and environmental information processing was higher in the rhizoplane and rhizosphere than in the endosphere (**Supplementary Figures S2D–F**).

### 3.5 Relationship between environmental factors and bacterial communities

**Figure 5:**
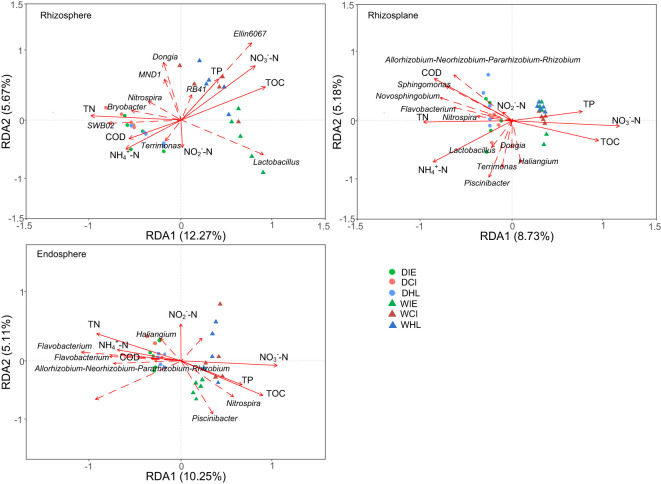
Redundancy analysis of the correlation between the environmental variables, dominant bacteria, and microbial communities in different root components (i.e. rhizosphere, rhizoplane, and endosphere). COD, chemical oxygen demand; TN, total nitrogen; TOC, total organic content; TP, total phosphorus; D, dry season; W, wet season; IE, *I. ensata*; CI, *C. indica*; HL, *H. littoralis*.

The RDA results revealed significant changes in the correlations between bacteria at the genus level within three plant species and environmental variables across different root components and seasons (**Figure 5**). In the dry season, certain bacteria (e.g., Bryobacter, SWB02, Sphingomonas and Terrimonas) were positively related to TN, COD, and NH_4_^+^-N in the rhizosphere, rhizoplane and Endosphere, respectively. Similarly, other bacteria (e.g., Allorhizobium, Sphingomonas, Nitrospira, Flavobacterium and Novosphingobium) and additional bacteria (e.g., Allorhizobium, Acidovorax, Hydrogenophaga, Flavobacterium and Haliangium) exhibited positive correlations with TN, COD, and NH_4_^+^-N in the rhizosphere, rhizoplane and Endosphere, respectively. While in the wet season, some bacteria communities (e.g., Ellin6067, Lactovacillus and RB41) were positively related to TP, TOC, and NO_3_^–^N in the rhizosphere while other bacteria (e.g., Nitrospira, Hyphomicrobium, and Piscinibacter) were positively related to TP, TOC and NO_3_^–^N in the Endosphere. These findings underscore the dynamic nature of bacterial correlations with environmental variables across different root components and seasonal conditions.

## 4 Discussion

### 4.1 Bacterial community composition and diversity were influenced by root, plant species and seasonality

The study highlighted that the characteristics of bacterial communities associated with roots were significantly affected by different plant species (*I. ensata*, *C. indica*, and *H. littoralis*), root components (rhizosphere, rhizoplane, and endosphere), and environmental factors (**Figures 2**, **3**). Across the rhizosphere, rhizoplane, and endosphere of three plant species, Proteobacteria, Cyanobacteria, Bacteroides, and Acidobacteria were predominantly components (**Figure 2**). The rhizosphere consistently exhibited the highest relative abundance of bacteria, aligning with the findings of Beckers et al. (2017) and Coleman et al. (2016). The widely acknowledged notion that the rhizosphere constitutes an environment that fosters more abundant bacterial populations compared to the non-rhizosphere soil further supports this observation (Qiao et al., 2017). Bacterial communities in the rhizosphere have the potential to translocate into plant tissues and establish residence in root endosphere, as demonstrated by previous studies (Hacquard et al., 2015; Vandenkoornhuyse et al., 2015). Within the same plant species, the alpha diversity of bacteria communities decreased from the rhizosphere to the endosphere. This decline may be caused by the selective effect exerted by the endosphere environment (Philippot et al., 2013). The influent sewage contains a high concentration of non-native bacterial species, and the plant’s immune mechanisms effectively eliminate numerous pathogenic bacteria (Mucyn et al., 2006). This process leads to a variation in microbial diversity between the rhizosphere and the endosphere. The selection process may be related to the expression of genes involved in various functions (Bulgarelli et al., 2012). Generally, the colonization of bacteria in different root components is primarily driven by two factors: (i) root rhizodeposits, including root exudates and mucilage of root caps, and (ii) the relatively simple or inelaborate chemo-attraction of the bacteria to the root exudates (Walker et al., 2003). These two factors may explain the α diversity were lower in *I. ensata* roots than in *C. indica* and *H. littoralis* roots (**Figure 3**). Specifically, *C. indica* and *H. littoralis* exhibited more extensive root systems compared to *I. ensata*, and they released greater amounts of oxygen and organic compounds to facilitate bacterial proliferation (Lee and Scholz, 2007). These emissions contributed to the enrichment of bacterial communities associated with their roots, increasing the bacterial diversity for *C. indica* and *H. littoralis*.

Unlike the α diversities of bacterial communities, the β diversities exhibited low variability during the dry season but demonstrated a high variability during the wet season within the rhizosphere (**Figure 4**). Bacteria, characterized by small size and a short life cycle, are inherently susceptible to disturbances from the external environment (Zhang et al., 2020). The polluted water releases a complex array of pollutant and microorganisms into the environment to affect the bacterial communities in the rhizosphere directly (McLellan et al., 2010), making bacterial composition became similar. However, this phenomenon was changed in wet season (**Figure 4**-Wet). The bacterial abundance and diversity increased significantly under the relatively high temperature and humidity in constructed wetland. Besides, environmental variables, specifically TOC, TP, and NH_4_^+^-N, provided nutrients and energy sources for different bacterial communities (**Figure 1**). More bacterial communities involved in the absorption and transformation of organic carbon, phosphorus, and nitrogen increased to meet the rapid growth of plants, which resulted in the similarity of bacterial communities in the endosphere.

### 4.2 Process of water purification in different root components

The numbers of bacterial biomarkers increased from the inside of the root system to the outside (i.e. from the endosphere to the rhizosphere) in the three plants (**Supplementary Figure S1**). The abundance and composition of biomarkers were used to characterize the level of pollutant degradation and to determine which microbial taxa are more sensitive to the external environment (Wu et al., 2023). Within the bacterial communities of the root components of *C. indica*, the community on the rhizoplane had the highest number of biomarkers, indicating that it was most sensitive to the external environment. This observation was consistent with the findings of Chen et al. (2020). The results of LEfSe further revealed differences in the dominated bacterial communities in root components, i.e. the number of primarily dominant bacterial communities in the rhizosphere was smaller for all three plants compared to the endosphere, and the bacterial composition was largely distinct, with each having an LDA score of > 4.0. Our results showed soil/water environment might be the main factor in changing the structure of the bacterial community, while it turned the equilibrium state in the root systems.

The content of TN, TP, TOC, COD, NO_4_^+^-N and NO_3_^−^-N in constructed wetland significantly decreased after being treated with plants, and the pollutant removal efficiency was higher in wet season (**Figure 1**). The consequence of pollutant removal efficiency was supported by previous research that both nitrification and denitrification would be inhibited strongly at temperatures below 20°C (Yao et al., 2013). Cyanobacteria, mainly Oxyphotobacteria, were significantly enriched in the rhizoplane of all three plant species (**Figure 2**; **Supplementary Figure S3**). Oxyphotobacteria, known for their strong ability to decompose and transform organic matter, can photosynthesize under anaerobic conditions and release oxygen that degrades nitrite in water, thereby enhancing water purification (Ren et al., 2023). The characteristic of Oxyphotobacteria of three plant species suggested that the catabolic degradation of contaminants occurred mainly in the rhizoplane. Proteobacteria were proven to reduce N and P levels in constructed wetlands by facilitating nutrient exchange and metabolite (Fu et al., 2006). For example, both Gammaproteobacteria and Deltaproteobacteria can removing nitrate and nitrite because they can fix N and perform denitrification under anaerobic conditions (Valaskova et al., 2009; Zhang et al., 2021). The abundance of Oxyphotobacteria, Gammaproteobacteria and Deltaproteobacteria were mainly in the root rhizosplane and endosphere, indicating that the process of water purification occurred primarily in the rhizosplane and endosphere. Dominant members of the root endosphere communities, such as Ignavibactera, Dependentiae, and Babeliales, have also been empirically proven to yield advantageous outcomes for plant growth metabolism and health (Innerebner et al., 2011). The distinct endosphere environment determined by the root selective absorption different plant species (Anisimov and Suslov, 2023), which also affected the structure of bacterial communities. Thus, we speculate that the water purification by bacterial communities depends on the different elements absorbed by the host plant’s immune system and is regulated by seasonality. This process mainly occurs in endosphere.

### 4.3 The potential functions of different bacterial communities determine the water purification capacity of plants

The potential functions of bacterial communities were different in different plant species and root components. The predominant bacterial genes were associated with metabolism, environmental information processing, and membrane transport in the rhizosphere and the rhizoplane (**Supplementary Figure S2**). Conversely, the predominant bacterial genes were related to metabolism in the endosphere. Metabolic functional genes in bacteria play a vital role in facilitating the uptake and utilization of amino acids, energy, carbohydrates, etc (Ruiz-Gonzalez et al., 2015). Bacteria reduce nitrate to nitrogen by denitrification (**Figure 5**). The bacterial communities with metabolic functions were predominantly preserved inside the roots, indicating that the main role of the bacteria in the roots was nutrient cycling (such as denitrification) and providing plant roots with the essential nutrients required for their growth. Compared to *C. indica* and *H. littoralis*, *I. ensata* preserved more metabolism pathways in all root components, indicative of high metabolically active (Chen et al., 2019). Genes associated with membrane transport were also important in the rhizosphere and rhizoplane of three plant species. With the increase in bacterial diversity, their genetic contributions facilitated the dissolution of iron and small molecules, as well as the transfer of dissolved oxygen (Wu et al., 2023; Ren et al., 2024). This process ensured and enhanced the absorption of minerals and amino acids from the rhizosphere and rhizoplane, promoted nutrient cycling within the endosphere, and ultimately influenced plant physiological processes.

The bacterial community demonstrated a positive correlation with TN, COD, and NO_4_^+^-N in the dry season (**Figure 5**). COD, containing heavy metals (HMs) and polycyclic aromatic hydrocarbons (PAHs), was significantly degraded by Bryobacter, Nitrospira, and Sphingobium (Liang et al., 2021; Yang et al., 2022). RB41 and MND1, functioning as module hubs, have important ecological roles in triggering resistance or tolerance to toxicity (Wang et al., 2019). RB41 and MND1 were positively related to *C. indica* in the rhizosphere (**Figure 5**), indicating bacterial communities enhanced stress resistance for *C. indica*. Thus, bacterial communities in dry season is to protect plants from toxic pollutants. In the wet season, the bacterial species composition was all positively related to TP, TOC, and NO_3_^–^N in three root components. The biomass of plants and abundance microbes increased in wet or hot seasons (Pang et al., 2016; Schofield et al., 2022). When the content of water pollutants is stable, the increase of microbial abundance can significantly enhance the transport of rhizosphere and the metabolic function of endosphere to promote plant growth (Makgato and Chirwa, 2020; Singh et al., 2025). Our predictions of gene functions indicate that microbial community ensure survival by removing pollutants in the dry season, while the microbial community expands in the wet season, achieving better pollutants removal efficiency.

## 5 Conclusion

The variations of composition, diversity, and functions of bacterial communities were largely attributed to root components (i.e. rhizosphere, rhizoplane, and endosphere), plant species and seasonality. In wet season, the similarity of bacterial communities in rhizosphere caused by water environment was changed. *I. ensata* was theoretically suggested superior water purification capabilities, while *C. indica* could handle multiple water pollutants among the three plant species. It became evident that the root interior, encompassing the rhizoplane and endosphere, is the main site of the water purification process. Bacterial population size directly affects the pollutant removal efficiency. Bacterial population size was small in dry season, mainly purifying pollutants and protect plant from damage; the bacterial populations expanded in wet season, mainly absorbing nutrients (e.g., TOC, N and P) to promote its own and plant growth. The co-cultivation of multiple plant species and elevation of water temperature appeared to be advantageous for enhancing water purification within constructed wetland systems. These findings underscore the importance of understanding the interplay between plant species, root components, and bacterial communities in optimizing water treatment processes.

## Data Availability

The original contributions presented in the study are publicly available. This data can be found here: NCBI, PRJNA1216410.

## References

[B1] Ali M. D. Ahmed T. Ibrahim E. Rizwan M. Chong K. P. Yong J. W. H. (2024). A review on mechanisms and prospects of endophytic bacteria in biocontrol of plant pathogenic fungi and their plant growth-promoting activities. Heliyon 10, e31573. doi: 10.1016/j.heliyon.2024.e31573 38841467 PMC11152693

[B2] Anisimov A. V. Suslov M. A. (2023). Measuring of water transport selectively along the plant root plasmodesmata using gradient nuclear magnetic resonance with paramagnetic doping. Plant Physiol. Biochem. 194, 263–270. doi: 10.1016/j.plaphy.2022.11.028 36442358

[B3] APHA (1998). Standard methods for the examination of water and wastewater (Washington DC: American Public Health Association).

[B4] Arroyo P. Ansola G. Miera L. E. S. D. (2013). Effects of substrate, vegetation and flow on arsenic and zinc removal efficiency and microbial diversity in constructed wetlands. Ecol. Eng. 51, 95–103. doi: 10.1016/j.ecoleng.2023.12.013

[B5] Balasooriya W. K. Denef K. Huygens D. Boeckx P. (2012). Translocation and turnover of rhizodeposit carbon within soil microbial communities of an extensive grassland ecosystem. Plant Soil 376, 61–73. doi: 10.1007/s11104-012-1343-z

[B6] Beach M. (2001). Water, pollution, and public health in China. Lancet 358, 735. doi: 10.1016/S0140-6736(01)05943-8 11551592

[B7] Beckers B. Op De Beeck M. Weyens N. Boerjan W. Vangronsveld J. (2017). Structural variability and niche differentiation in the rhizosphere and endosphere bacterial microbiome of field-grown poplar trees. Microbiome 5, 25. doi: 10.1007/s42770-020-00337-7 28231859 PMC5324219

[B8] Bokulich N. A. Subramanian S. Faith J. J. Gevers D. Gordon J. I. Knight R.. (2013). Quality-filtering vastly improves diversity estimates from Illumina amplicon sequencing. Nat. Methods 10, 57–59. doi: 10.1038/nmeth.2276 23202435 PMC3531572

[B9] Bolger A. M. Lohse M. Usadel B. (2014). Trimmomatic: a flexible trimmer for Illumina sequence data. Bioinf 30, 2114–2120. doi: 10.1093/bioinformatics/btu170 PMC4103590 24695404

[B10] Brisson J. Chazarenc F. (2009). Maximizing pollutant removal in constructed wetlands: should we pay more attention to macrophyte species selection? Sci. Total Environ. 407, 3923–3930. doi: 10.1016/j.scitotenv.2008.05.047 18625516

[B11] Bulgarelli D. Rott M. Schlaeppi K. Ver Loren Van Themaat E. Ahmadinejad N. Assenza F.. (2012). Revealing structure and assembly cues for Arabidopsis root-inhabiting bacterial microbiota. Nature 488, 91–95. doi: 10.1038/nature11336 22859207

[B12] Chen L. Brookes P. C. Xu J. Zhang J. Zhang C. Zhou X.. (2016). Structural and functional differentiation of the root-associated bacterial microbiomes of perennial ryegrass. Soil Biol. Biochem. 98, 1–10. doi: 10.1016/j.soilbio.2016.04.004

[B13] Chen J. Wu Q. Li S. Ge J. Fuhrmann J. J. (2019). Diversity and function of soil bacterial communities in response to long-term intensive management in a subtropical bamboo forest. Geoderma 354, 113894. doi: 10.1016/j.geoderma.2019.113894

[B14] Chen J. Xu D. Chao L. Liu H. Bao Y. (2020). Microbial assemblages associated with the rhizosphere and endosphere of an herbage, *Leymus chinensis* . Microb. Biotechnol. 13, 1390–1402. doi: 10.1111/1751-7915.13558 32227622 PMC7415361

[B15] Coleman D. D. Desgarennes D. Fonseca G. C. Gross S. Clingenpeel S. Woyke T.. (2016). Plant compartment and biogeography affect microbiome composition in cultivated and native Agave species. New Phytol. 209, 798–811. doi: 10.1111/nph.13697 26467257 PMC5057366

[B16] Edgar R. C. (2013). UPARSE: highly accurate OTU sequences from microbial amplicon reads. Nat. Methods 10, 996–1001. doi: 10.1038/nmeth.2604 23955772

[B17] Edgar R. C. Haas B. J. Clemente J. C. Quince C. Knight R. (2011). UCHIME: improves sensitivity and speed of chimera detection. Bioinf 27, 2194–2200. doi: 10.1093/bioinformatics/btr381 PMC3150044 21700674

[B18] Edwards J. Johnson C. Santos-Medellin C. Lurie E. Podishetty N. K. Bhatnagar S.. (2015). Structure, variation, and assembly of the root-associated microbiomes of rice. Proc. Natl. Acad. Sci. U.S.A. 112, E911–E920. doi: 10.1073/pnas.1414592112 25605935 PMC4345613

[B19] Fu R. B. Yang H. Z. Gu G. W. Zhang Z. (2006). Analysis of substrate microorganisms status in constructed wetlands and their correlation with pollutants removal for wastewater treatment. Environ. Sci. Res. 18, 44–49. doi: 10.1007/s10971-005-6694-y

[B20] Gao Z. Song J. M. Pan C. P. (2020). Application of constructed wetland in comprehensive project of Shenzhen Pingshan river. Water supply drainage China 36, 65–68. doi: 10.19853/j.zgjsps.1000-4602.2020.02.012

[B21] Guo Y. Gong H. Guo X. (2015). Rhizosphere bacterial community of Typha angustifolia L. and water quality in a river wetland supplied with reclaimed water. Appl. Microbiol. Biotech. 99, 2883–2893. doi: 10.1007/s00253-014-6182-9 25412576

[B22] Hacquard S. Garrido-Oter R. Gonzalez A. Spaepen S. Ackermann G. Lebeis S.. (2015). Microbiota and host nutrition across plant and animal kingdoms. Cell Host Microbe 17, 603–616. doi: 10.1016/j.chom.2015.04.009 25974302

[B23] Imfeld G. Braeckevelt M. Kuschk P. Richnow H. H. (2009). Monitoring and assessing processes of organic chemicals removal in constructed wetlands. Chemosphere 74, 349–362. doi: 10.1016/j.chemosphere.2008.09.062 18996559

[B24] Innerebner G. Knief C. Vorholt J. A. (2011). Protection of Arabidopsis thaliana against leaf-pathogenic Pseudomonas syringe by Sphingomonas strains in a controlled model system. Appl. Environ. Microbiol. 77, 3202–3210. doi: 10.1128/AEM.00133-11 21421777 PMC3126462

[B25] Kovacs G. Burghardt J. Pradella S. Schumann P. Stackebrandt E. Mhrialigeti K. (1999). Kocuria palustris sp. nov. and Kocuria rhizophila sp. nov., isolated from the rhizoplane of the narrow-leaved cattail (*Typha angustifolia*). Int. J. Syst. Bacteriol 49, 167–173. doi: 10.1099/00207713-49-1-167 10028258

[B26] Langille M. G. Zaneveld J. Caporaso J. G. Mcdonald D. Knights D. Reyes J. A.. (2013). Predictive functional profiling of microbial communities using 16S rRNA marker gene sequences. Nat. Biotechnol. 31, 814–821. doi: 10.1038/NBT.2676 23975157 PMC3819121

[B27] Lee B. H. Scholz M. (2007). What is the role of Phragmites australis in experimental constructed wetland filters treating urban runoff? Ecolol. Eng. 29, 87–95. doi: 10.1016/j.ecoleng.2006.08.001

[B28] Liang J. Q. Xu J. T. Zhao W. J. Wang J. F. Chen K. Li Y. Q.. (2021). Benzo[a]pyrene might be transported by a TonB-dependent transporter in *Novosphingobium pentaromativorans* US6-1. J. Hazard. Mater 404, 124037. doi: 10.1016/j.hazmat.2020.124037 33059256

[B29] Liao D. Huang H. Zhuang S. Hong Y. W. (2018). Effects of exotic Spartina alterniflora on rhizosphere and endophytic bacterial community structures and diversity in roots of native mangroves. Chin. J. Appl. Environ. Biol. 24, 269–275. doi: 10.19675/j.cnki.1006-687x.2017.04032

[B30] Liu F. F. Fan J. Du J. Shi X. Zhang J. Shen Y. (2019). Intensified nitrogen transformation in intermittently aerated constructed wetlands: Removal pathways and microbial response mechanism. Sci. Total Environ. 650, 2880–2887. doi: 10.1016/j.scitotenv.2018.10.037 30373064

[B31] Liu D. Ge Y. Chang J. Peng C. Gu B. Chan G. Y. S.. (2008). Constructed wetlands in China: recent developments and future challenges. Front. Ecol. Environ. 7, 261–268. doi: 10.1890/070110

[B32] Liu W. J. Xu X. Y. He H. Zhu D. L. Xu R. C. Sun C. (2016). Comparison of treatment performances of simulated urban water in constructed wetlands planted with four types of wetland plant. J. Environ. Eng. 10, 6313–6319. doi: 10.12030/j.cjee.201505212

[B33] Lu J. Guo Z. Kang Y. Fan J. Zhang J. (2020). Recent advances in the enhanced nitrogen removal by oxygen-increasing technology in constructed wetlands. Ecotoxicol. Environ. Saf. 205, 111330. doi: 10.1016/j.ecoenv.2020.111330 32977288

[B34] Magoc T. Salzberg S. L. (2011). FLASH: fast length adjustment of short reads to improve genome assemblies. Bioinf 27, 2957–2963. doi: 10.1093/bioinformatics/btr507 PMC3198573 21903629

[B35] Makgato S. S. Chirwa E. M. N. (2020). The desulphurization potential of Waterberg steam coal using bacteria isolated from coal: The SO_2_ emissions control technique. J. Clean. Prod 263, 121051. doi: 10.1016/j.jclepro.2020.121051

[B36] Man Y. Wang J. Tam N. F. Wan X. Huang W. Zheng Y.. (2020). Responses of rhizosphere and bulk substrate microbiome to wastewater-borne sulfonamides in constructed wetlands with different plant species. Sci. Total Environ. 706, 135955. doi: 10.1016/j.scitotenv.2019.135955 31855648

[B37] Mathavarajah S. Stoddart A. K. Gagnon G. A. Dellaire G. (2020). Pandemic danger to the deep: The risk of marine mammals contracting SARS-CoV-2 from wastewater. Sci. Total Environ. 760, 143346. doi: 10.1016/j.scitotenv.2020.143346 33160659 PMC7598747

[B38] McLellan S. L. Huse S. M. Mueller-Spitz S. R. Andreishcheva E. N. Sogin M. L. (2010). Diversity and population structure of water-derived microorganisms in wastewater treatment plant influent. Environ. Microbiol. 12, 378–392. doi: 10.1111/j.1462-2920.2010.02204.x 19840106 PMC2868101

[B39] Mitter E. K. De Freitas J. R. Germida J. J. (2017). Bacterial root microbiome of plants growing in oil sands reclamation covers. Front. Microbiol. 8. doi: 10.3389/fmicb.2017.00849 PMC5432656 28559882

[B40] Montreemuk J. Stewart T. N. Prapagdee B. (2023). Bacterial-assisted phytoremediation of heavy metals: Concepts, current knowledge, and future directions. Environ. Technol. Inno 33, 103488. doi: 10.1016/j.eti.2023.103488

[B41] Morris S. J. Blackwood C. B. (2015). “Chapter 10 - the ecology of the soil biota and their function,” in Soil microbiology, ecology and biochemistry (Fourth edition), 273–309. doi: 10.1016/B978-0-12-415955-6.00010-4

[B42] Mucyn T. S. Clemente A. Andriotis V. M. E. Balmuth A. L. Oldroyd G. E. D. Staskawicz B. J.. (2006). The tomato NBARC-LRR protein prf Interacts with pto kinase *in vivo* to regulate specific plant immunity. Plant Cell 18, 2792–2806. doi: 10.1105/tpc.106.044016 17028203 PMC1626632

[B43] Naylor D. Degraaf S. Purdom E. Coleman-Derr D. (2017). Drought and host selection influence bacterial community dynamics in the grass root microbiome. ISME J. 11, 2691–2704. doi: 10.1038/ismej.2017.118 28753209 PMC5702725

[B44] Pang S. Zhang S. Lv X. Han B. Liu K. Qiu C.. (2016). Characterization of bacterial community in biofilm and sediments of wetlands dominated by aquatic macrophytes. Ecol. Eng. 97, 242–250. doi: 10.1016/j.ecoleng.2016.10.011

[B45] Pei Y. Yu Z. Ji J. Khan A. Li X. (2018). Microbial community structure and function indicate the severity of chromium contamination of the yellow river. Front. Microbiol. 9. doi: 10.3389/fmicb.2018.00038 PMC5810299 29472897

[B46] Philippot L. Raaijmakers J. M. Lemanceau P. van der Putten W. H. (2013). Going back to the roots: the microbial ecology of the rhizosphere. Nat. Rev. Microbiol. 11, 789–799. doi: 10.1038/nrmicro3109 24056930

[B47] Qiao J. Xu X. Liang X. Liu Y. Borriss R. Liu Y. (2017). Addition of plant-growth-promoting Bacillus subtilis PTS-394 on tomato rhizosphere has no durable impact on composition of root microbiome. BMC Microbiol. 17, 131. doi: 10.1186/s12866-017-1039-x 28583081 PMC5460418

[B48] Quast C. Pruesse E. Yilmaz P. Gerken J. Schweer T. Yarza P.. (2013). The SILVA ribosomal RNA gene database project: improved data processing and web-based tools. Nucleic Acids Res. 41, 590–596. doi: 10.1093/nar/gks1219 PMC3531112 23193283

[B49] Ren X. Li J. Zhou Z. Zhang Y. Wang Z. Zhang D.. (2023). Impact of invertebrates on water quality safety and their sheltering effect on bacteria in water supply systems. Environ. Pollu. 330, 121750. doi: 10.1016/j.envpol.2023.121750 37149252

[B50] Ren T. Jin X. Deng S. Guo K. Gao Y. Shi X.. (2024). Oxygen sensing regulation mechanism of Thauera bacteria in simultaneous nitrogen and phosphorus removal process. J. Clean Prod 434, 140332. doi: 10.1016/j.jclepro.2023.140332

[B51] Rifaat H. M. Márialigeti K. Kovács G. (2002). Investigations on rhizoplane actinobacteria ommunities of papyrus (*Cyperus papyrus*) from an Egyptian wetland. Acta Microbiologica Immunologica Hungarica 49, 423–432. doi: 10.1556/AMicr.49.2002.4.1 12512252

[B52] Ruiz-Gonzalez C. Nino-Garcia J. P. Del Giorgio P. A. (2015). Terrestrial origin of bacterial communities in complex boreal freshwater networks. Ecol. Lett. 18, 1198–1206. doi: 10.1111/ele.12499 26306742

[B53] Saeed T. Paul B. Afrin R. Al-Muyeed A. Sun G. (2016). Floating constructed wetland for the treatment of polluted river water: A pilot scale study on seasonal variation and shock load. Chem. Eng. J. 287, 62–73. doi: 10.1016/j.cej.2015.10.118

[B54] Schofield B. J. Andreani N. A. Günther C. S. Law G. R. McMahon G. Swainson M.. (2022). Livestock microbial landscape patterns: Retail poultry microbiomes significantly vary by region and season. Food Microbiol. 101, 103878. doi: 10.1016/j.fm.2021.103878 34579846 PMC8494115

[B55] Shao J. He Y. Zhang H. Chen A. Lei M. Chen J.. (2016). Silica fertilization and nano-MnO(2) amendment on bacterial community composition in high arsenic paddy soils. Appl. Microbiol. Biotechnol. 100, 2429–2437. doi: 10.1007/s00253-015-7131-y 26563550

[B56] Sims A. Gajaraj S. Hu Z. (2012). Seasonal population changes of ammonia-oxidizing organisms and their relationship to water quality in a constructed wetland. Ecol. Eng. 40, 100–107. doi: 10.1016/j.ecoleng.2011.12.021

[B57] Singh S. Suman S. K. Dutta K. Daverey A. (2025). Comparison of *Canna indica* and Acorus calamus for surfactant removal in biochar augmented constructed wetlands. Environ. Chem. Ecotoxicol 7, 130–140. doi: 10.1016/j.enceco.2024.11.003

[B58] Sun X. Song B. Xu R. Zhang M. Gao P. Lin H.. (2021). Root-associated (rhizosphere and endosphere) microbiomes of the *Miscanthus sinensis* and their response to the heavy metal contamination. J. Environ. Sci. 104, 387–398. doi: 10.1016/j.jes.2020.12.019 33985741

[B59] Teurlincx S. Kuiper J. J. Hoevenaar E. C. M. Lurling M. Brederveld R. J. Veraart A. J.. (2019). Towards restoring urban waters: understanding the main pressures. Curr. Opin. Environ. Sust 36, 49–58. doi: 10.1016/j.cosust.2018.10.011

[B60] Thurston J. A. Foster K. E. Karpiscak M. M. Gerba C. P. (2001). Fate of indicator microorganisms, giardia and cryptosporidium in subsurface flow constructed wetlands. Water Res. 35, 1547–1551. doi: 10.1016/S0043-1354(00)00414-0 11317902

[B61] Valaskova V. de Boer W. Gunnewiek P. J. Pospisek M. Baldrian P. (2009). Phylogenetic composition and properties of bacteria coexisting with the fungus Hypholoma fasciculare in decaying wood. ISME J. 3, 1218–1221. doi: 10.1038/ismej.2009.64 19516282

[B62] Vandenkoornhuyse P. Quaiser A. Duhamel M. Van A. L. Dufresne A. (2015). The importance of the microbiome of the plant holobiont. New Phytol. 206, 1196–1206. doi: 10.1111/nph.13312 25655016

[B63] Vymazal J. (2014). Constructed wetlands for treatment of industrial wastewaters: A review. Ecol. Eng. 73, 724–751. doi: 10.1016/j.ecoleng.2014.09.034

[B64] Walker T. S. Bais H. P. Grotewold E. Vivanco J. M. (2003). Update on root exudation and rhizosphere biology: root exudation and rhizosphere biology. Plant Physiol. 132, 44–51. doi: 10.1104/pp.102.019661 12746510 PMC1540314

[B65] Wang M. Chen S. Chen L. Wang D. (2019). Responses of soil microbial communities and their network interactions to saline-alkaline stress in cd-contaminated soils. Environ. Pollu. 252, 1609–1621. doi: 10.1016/j.envpol.2019.06.082 31284203

[B66] Wang N. Zhu X. Zuo Y. Liu J. Yuan F. Guo Z.. (2023). Microbial mechanisms for methane source-to-sink transition after wetland conversion to cropland. Geoderma 429, 116229. doi: 10.1016/j.geoderma.2022.116229

[B67] Wang Q. Garrity G. M. Tiedje J. M. Cole J. R. (2007). Naive Bayesian classifier for rapid assignment of rRNA sequences into the new bacterial taxonomy. Appl. Environ. Microbiol. 73, 5261–5267. doi: 10.1128/AEM.00062-07 17586664 PMC1950982

[B68] Wang B. Zheng X. Zhang H. Yu X. Lian Y. Yang X.. (2021). Metagenomic insights into the effects of submerged plants on functional potential of microbial communities in wetland sediments. Mar. Life Sci. Tech 3, 405–415. doi: 10.1007/s42995-021-00100-3 PMC10077182 37073260

[B69] Wright E. S. Vetsigian K. H. (2016). Quality filtering of Illumina index reads mitigates sample cross-talk. BMC Genomics 17, 876. doi: 10.1186/s12864-016-3217-x 27814679 PMC5097354

[B70] Wu S. Wallace S. Brix H. Kuschk P. Kirui W. K. Masi F.. (2015). Treatment of industrial effluents in constructed wetlands: challenges, operational strategies and overall performance. Environ. pollut. 201, 107–120. doi: 10.1016/j.envpol.2015.03.006 25792030

[B71] Wu C. Wang M. Wang C. Zhao X. Liu Y. Masoudi A.. (2023). Reed biochar improved the soil functioning and bacterial interactions: A bagging experiment using the plantation forest soil (*Fraxinus chinensis*) in the Xiong’an new area, China. J. Clean Prod 410, 137316. doi: 10.1016/j.jclepro.2023.137316

[B72] Yang Z. N. Liu Z. S. Wang K. H. Liang Z. L. Abdugheni R. Huang Y.. (2022). Soil microbiomes divergently respond to heavy metals and polycyclic aromatic hydrocarbons in contaminated industrial sites. Environ. Sci. Ecotechnol 10, 100169. doi: 10.1016/j.ese.2022.100169 36159729 PMC9488039

[B73] Yao S. Ni J. Ma T. Li C. (2013). Heterotrophic nitrification and aerobic denitrification at low temperature by a newly isolated bacterium, Acinetobacter sp. HA2. Bioresource Technol. 139, 80–86. doi: 10.1016/j.biortech.2013.03.189 23644073

[B74] Yu G. Chen H. Chen J. Chen S. Long Y. Huang J.. (2024). Enhanced nitrogen removal through aerobic denitrifying bacteria in horizontal subsurface flow constructed wetlands: Influencing factors and microbial community structure. Chem. Eng. J. 481, 148654. doi: 10.1016/j.cej.024.148654

[B75] Zhang Q. Chang Y. Liu B. Zhu H. (2021). Field assessment of full-scale solar-powered floating biofilm reactors for improving water quality in a micro-polluted river near Lake Taihu. J. Clean Prod 312, 127762. doi: 10.1016/j.jclepro.2021.127762

[B76] Zhang Q. Chen X. Zhang Z. Luo W. Wu H. Zhang L.. (2020). Performance and microbial ecology of a novel moving bed biofilm reactor process inoculated with heterotrophic nitrification-aerobic denitrification bacteria for high ammonia nitrogen wastewater treatment. Bioresource Technol. 315, 123813. doi: 10.1016/j.biortech.2020.123813 32702578

[B77] Zhang Y. Xu J. Riera N. Jin T. Li J. Wang N. (2017). Huanglongbing impairs the rhizosphere-to-rhizoplane enrichment process of the citrus root-associated microbiome. Microbiome 5, 97. doi: 10.1186/s40168-017-0304-4 28797279 PMC5553657

