# Supplementary material for: Functionality of bacterial communities in constructed wetlands used for water purification: influence of root components and seasonality
Source: Front Plant Sci. 2025 Feb 11;16:1480099. doi: 10.3389/fpls.2025.1480099 (PMC11850325; doi:10.3389/fpls.2025.1480099)
Supplement: Supplementary file 1 [file DataSheet1.docx]

*Supplementary Materials for*

**The structural and functional differentiation of root-associated bacterial communities as indicators for sewage purification in constructed wetlands**


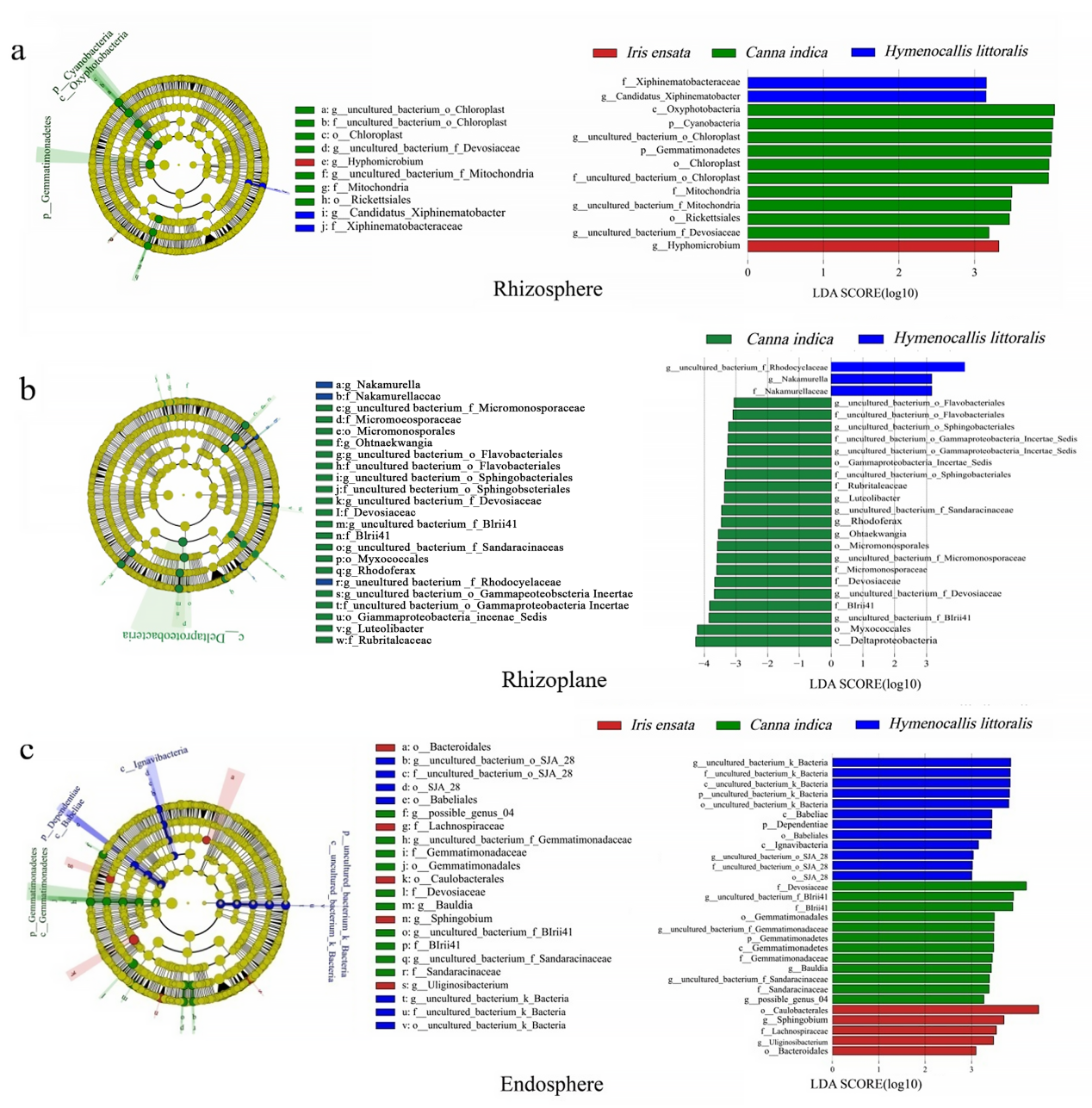


Fig. S1. Linear discriminant analysis effect size (LEfSe) analysis of the phylogenetic clade and LDA analysis chart of bacterial communities in the root compartments of the three plant species. (a) the differences of biomakers in rhziosphere of three plant species; (b) the differences of biomakers in rhzioplane of three plant species; (c) the differences of biomakers in endosphere of three plant species.


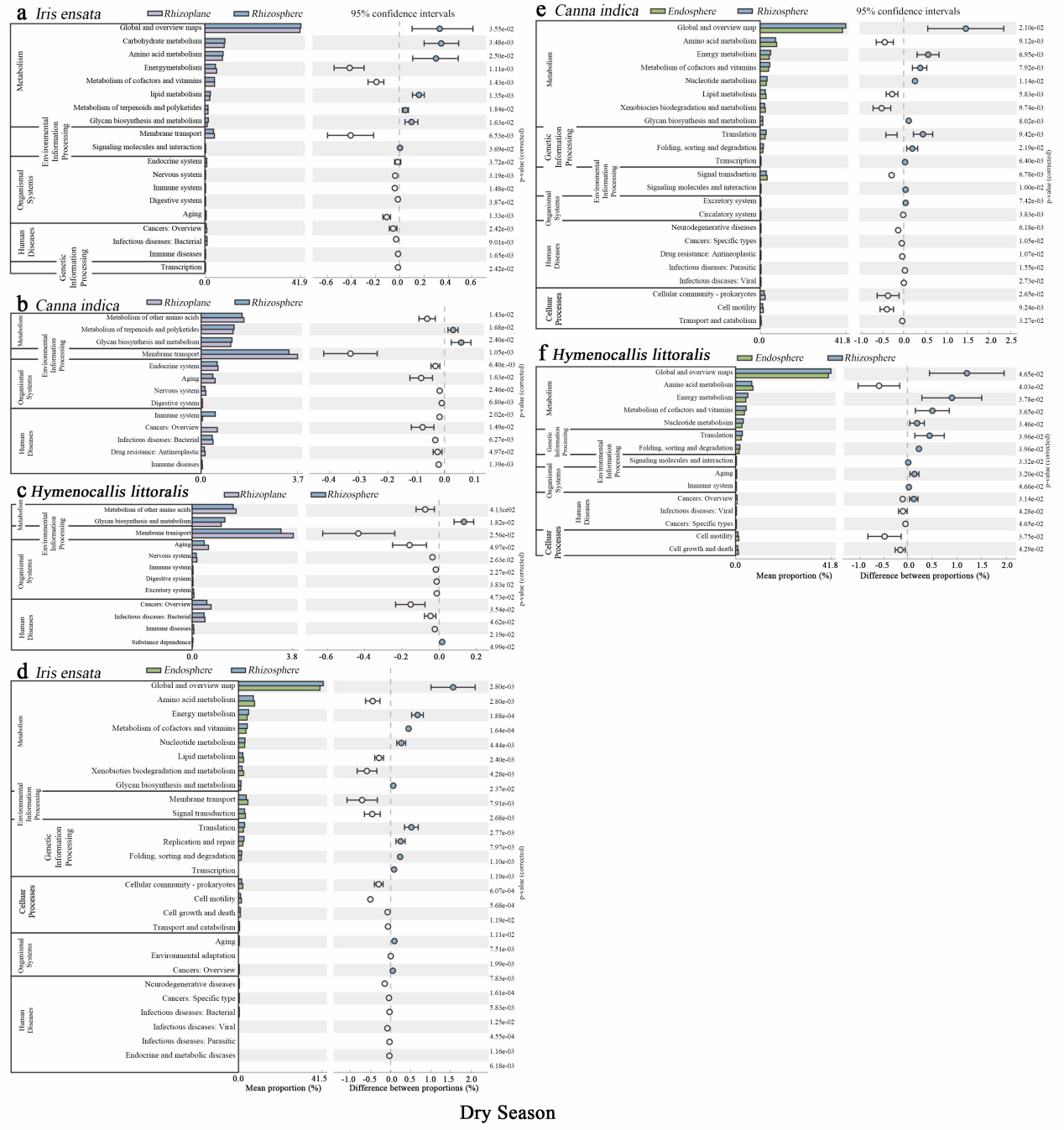


Fig. S2. Comparison of bacterial community functions predicted by PICRUSt in the root compartments of the three plant species in dry season. Figure (a) and (d) were the different function genes of root compartments in *Iris ensata*; Figure (b) and (e) were the different function genes of root compartments in *Canna indica*; Figure (c) and (f) were the different function genes of root compartments in *Hymenocallos littoralis*.


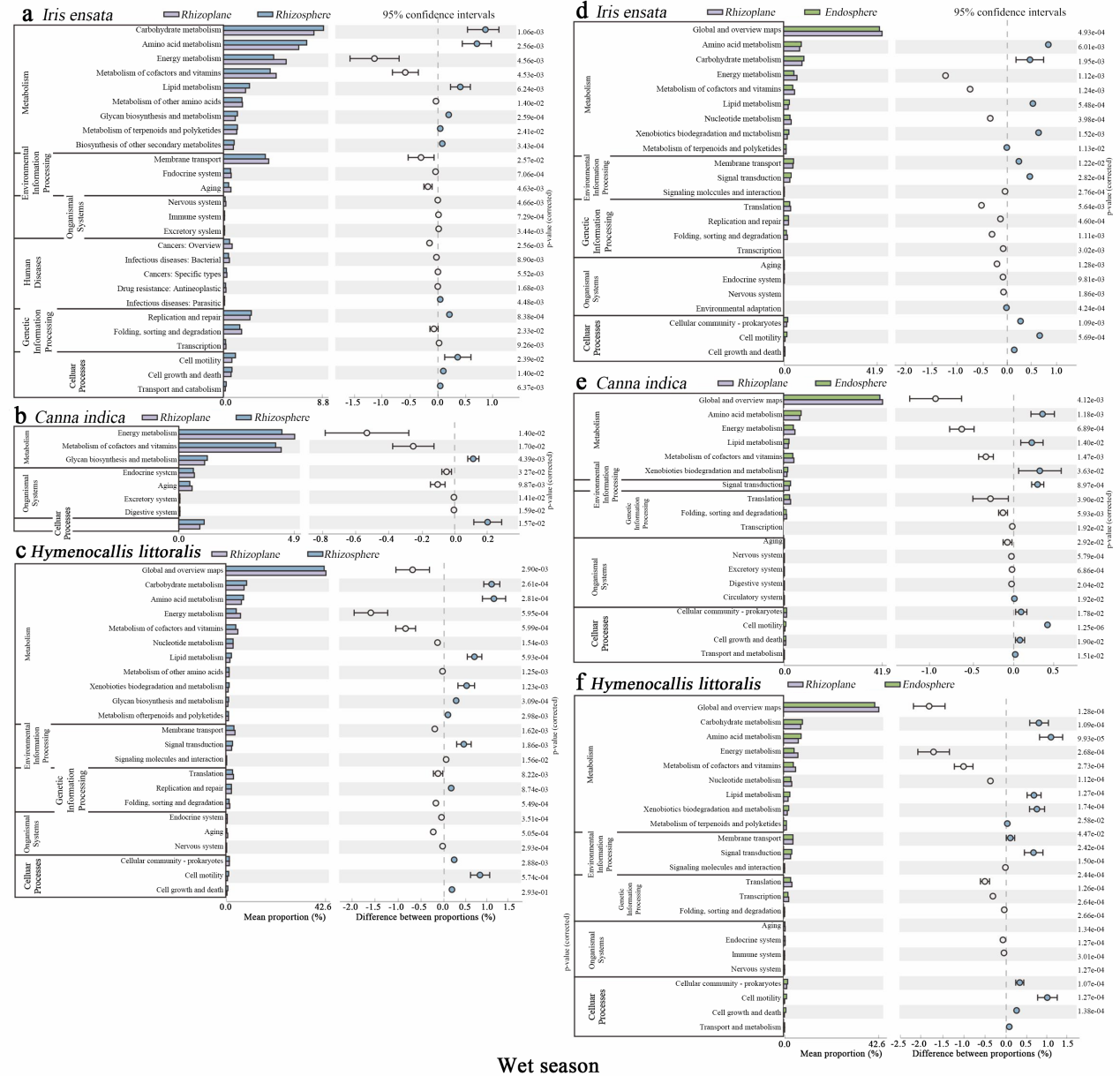


Fig. S3. Comparison of bacterial community functions predicted by PICRUSt in the root compartments of the three plant species in wet season. Figure (a) and (d) were the different function genes of root compartments in *Iris ensata*; Figure (b) and (e) were the different function genes of root compartments in *Canna indica*; Figure (c) and (f) were the different function genes of root compartments in *Hymenocallos littoralis*.


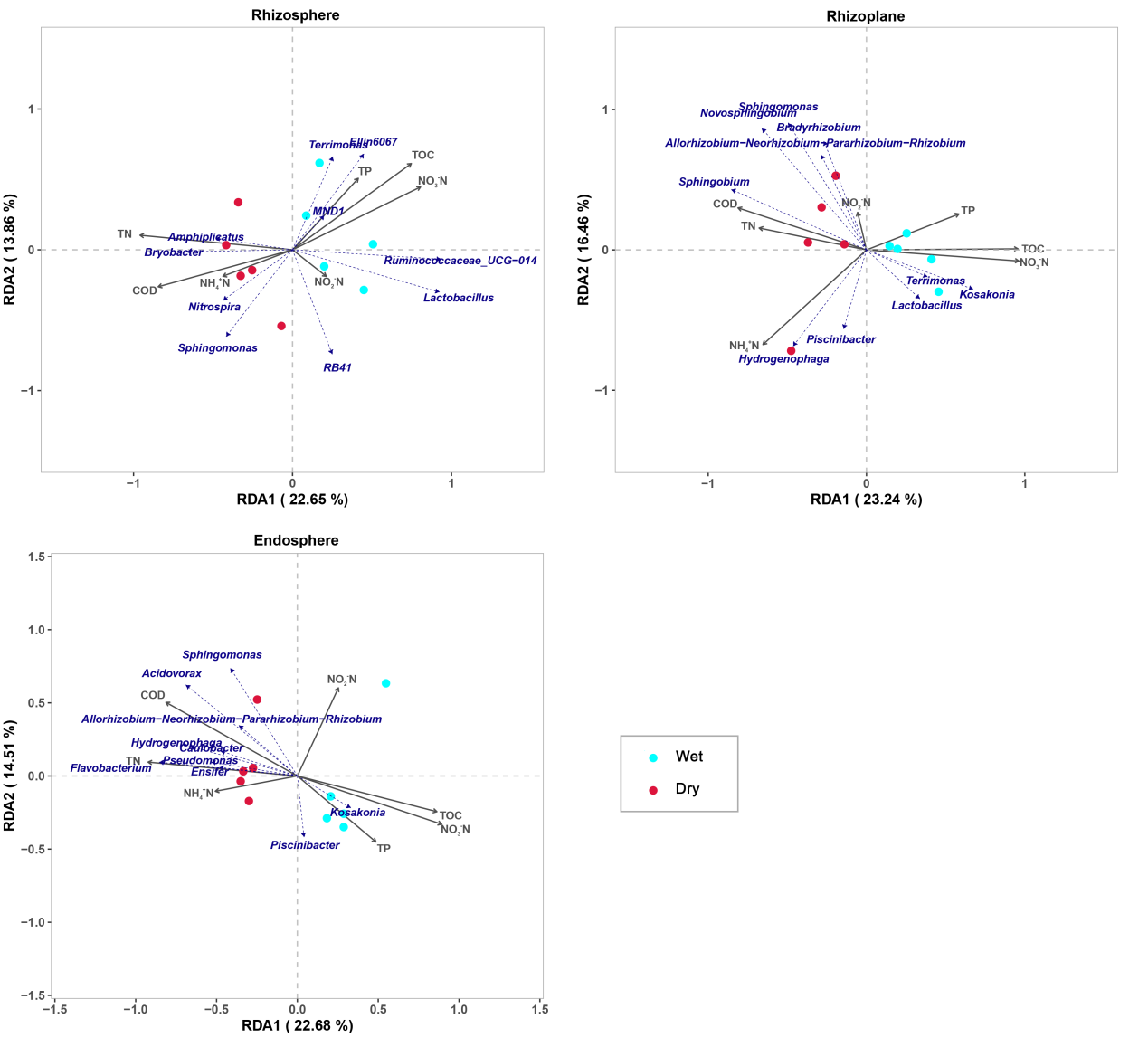


Fig. S4. the redundancy analysis of the correlation between the environmental variables, dominant bacteria, and microbial communities in different root components of *Iris ensata*.


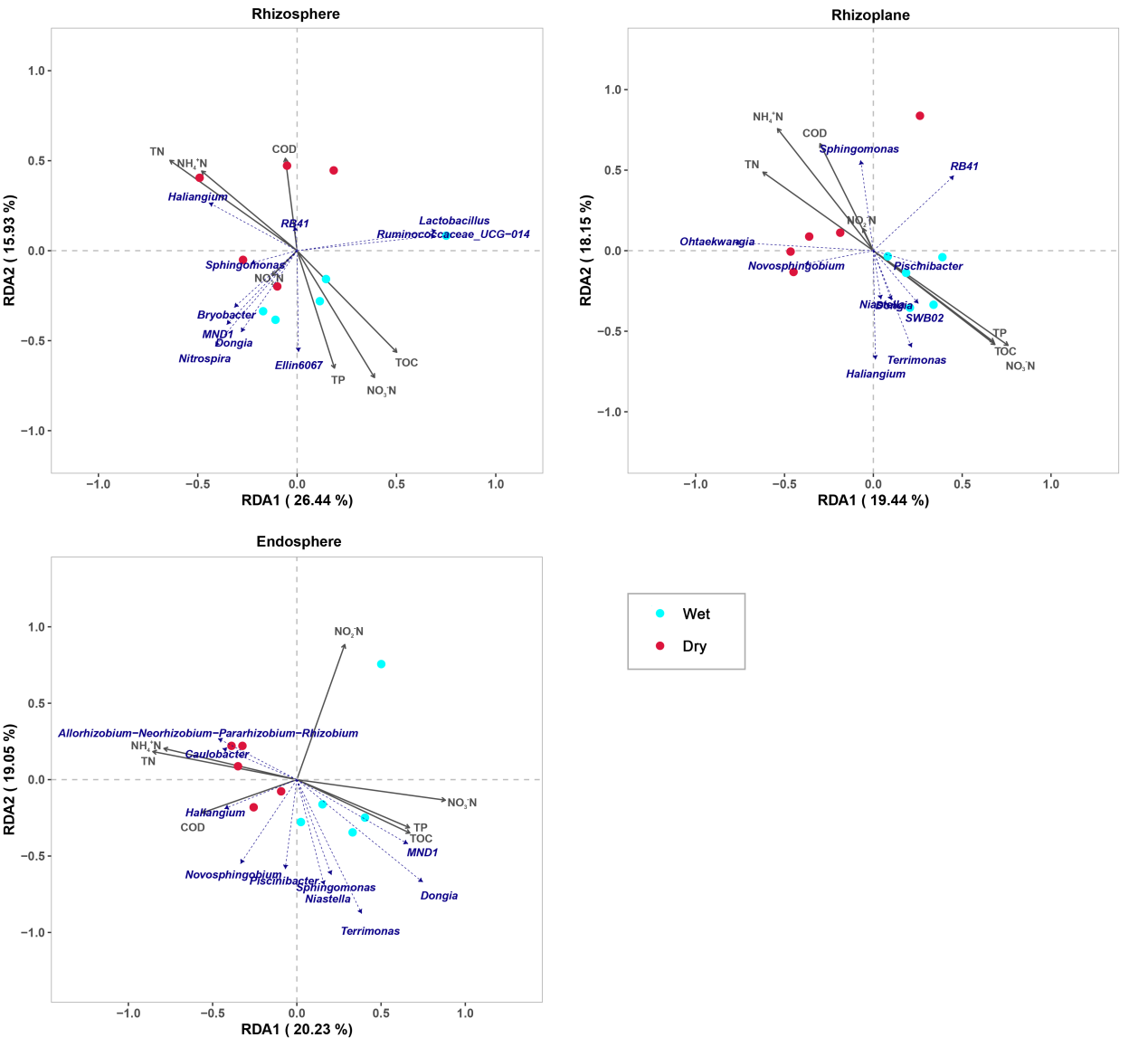


Fig. S5. the redundancy analysis of the correlation between the environmental variables, dominant bacteria, and microbial communities in different root components of *Canada indica*.


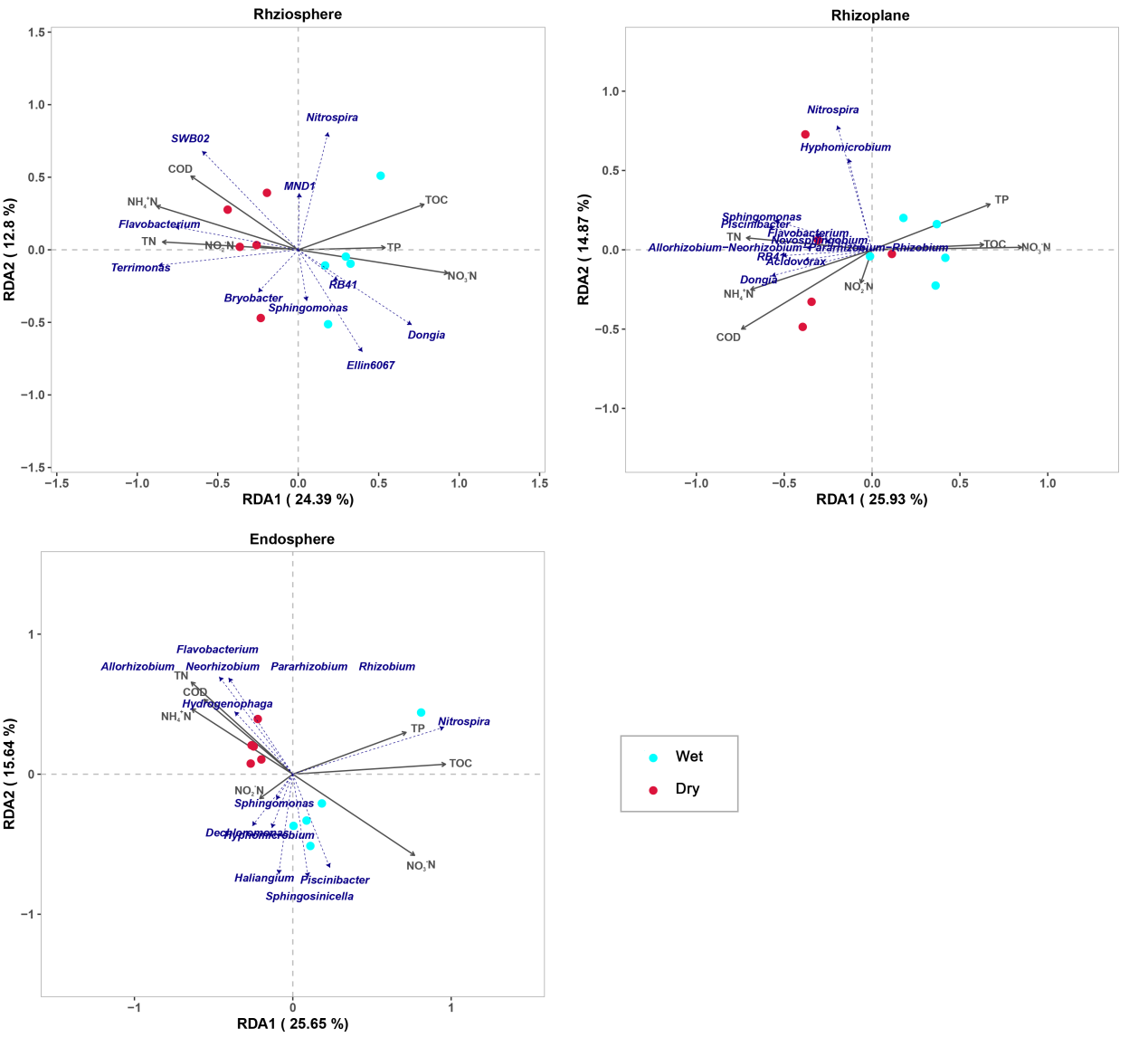


Fig. S6. the redundancy analysis of the correlation between the environmental variables, dominant bacteria, and microbial communities in different root components of *Hymenocallis littoralis*.

Table S1 Overview of water quality of water intake of experimental sample ponds in 2021. COD = chemical oxygen demand; TOC = total organic carbon; TN = total nitrogen; TP = total phosphorus.

| Index | NO_2_^-^-N (mg·L^-1^) | NO_3_^-^-N (mg·L^-1^) | NH_4_^+^-N (mg·L^-1^) | COD (mg·L^-1^) | TOC (mg·L^-1^) | TN (mg·L^-1^) | TP (mg·L^-1^) |
| --- | --- | --- | --- | --- | --- | --- | --- |
|  | 0.21±0.01 | 11.5±0.36 | 0.61±0.14 | 1.1±0.09 | 4.84±0.13 | 15.45±0.07 | 0.04±0.01 |

Table S2 The basic information about microbial data of three plant species.

| Root components of three plant species | *Iris ensata* | | | *Canada indica* | | | *Hymenocallis littoralis* | | |
| --- | --- | --- | --- | --- | --- | --- | --- | --- | --- |
|  | rhizosphere | rhizoplane | endosphere | rhizosphere | rhizoplane | endosphere | rhizosphere | rhizoplane | endosphere |
| OTU number | 2087 | 2020 | 1349 | 2158 | 2129 | 1673 | 2143 | 2103 | 1638 |
| Biomaker number | 1 | 0 | 5 | 10 | 21 | 12 | 2 | 3 | 12 |

Table S3 The relative abundance (%) of functional gene predicted by PICRUSt.

| Functions | Cellular Processes | Environmental Information Processing | Genetic Information Processing | Human Diseases | Metabolism | Organismal Systems |
| --- | --- | --- | --- | --- | --- | --- |
| Relative abundance (%) | 3.62 | 6.41 | 7.06 | 3.01 | 78.42 | 1.48 |
